# Maternal Pre-Pregnancy Nutritional Status and Physical Activity Levels and a Sports Injury Reported in Children: A Seven-Year Follow-Up Study

**DOI:** 10.3390/nu14040870

**Published:** 2022-02-18

**Authors:** Mario Kasović, Lovro Štefan, Pavel Piler, Martin Zvonar

**Affiliations:** 1 Department of General and Applied Kinesiology, Faculty of Kinesiology, University of Zagreb, 10000 Zagreb, Croatia; mario.kasovic@kif.hr; 2 Department of Sport Motorics and Methodology in Kinanthropology, Faculty of Sports Studies, Masaryk University, 61 137 Brno, Czech Republic; 3 RECETOX, Faculty of Science, Masaryk University, Kotlarska 2, 61 137 Brno, Czech Republic; pavel.piler@recetox.muni.cz (P.P.); zvonar@fsps.muni.cz (M.Z.)

**Keywords:** body mass index, physical activity, sports injury, longitudinal analysis, children

## Abstract

Objective: Our aim was to analyze dose–response associations between maternal pre-pregnancy body mass index and physical activity levels with childhood sports injury rates. Methods: Participants included pre-pregnant mothers (*n* = 4811) and their children at the age of 7 years (*n* = 3311). Maternal anthropometry (height, weight, and body mass index), time spent in physical activity, and education level were recorded. All sports injuries were defined as injuries reported in the past year by the children at the age of 7 years. Results: Children whose mothers were overweight/obese in the pre-pregnancy period were 2.04 (OR = 2.04, 95% CI = 1.12–3.71) times more likely to report a sports injury at the age of 7 years. Underweight mothers exhibited a 74% decrease in the odds of their children reporting a sports injury at follow-up (OR = 0.26, 95% CI = 0.10–0.68). Finally, an increase in maternal physical activity across the last three quartiles was associated with a lower odds of sports injuries. Conclusions: The risk of reporting a sports injury was greater for children whose mothers were overweight/obese in the pre-pregnancy period. However, there was a lower risk with both maternal underweight status and increasing minutes of physical activity.

## 1. Introduction

Sports participation rates in the Czech Republic are relatively high [1]. Estimates suggest that 63% of Czech Republic children and youth participate in organized sports or physical activity programs [1]. Moreover, 80% of schools have school policies concerning physical activity and 89% offer physical activity opportunities regularly [1]. Although the health benefits of regular physical activity are well-documented [2,3], studies have shown that higher levels of physical activity and participation in sports are accompanied by an inherent risk of a sports injury [4,5,6]. In some countries, sports injuries in youth are the leading cause of injury [7], with an estimated incidence proportion of injury, based on the previous year, of 62% [8]. Evidence shows that promoting physical activity and sports participation in youth with obesity may increase the risk of an injury occurring [8,9,10,11]. Thus, both high physical activity and body mass index serve as risk factors of a sports injury.

Cross-sectional [8,9,10,11] and longitudinal [4] studies have examined the associations between physical activity, body mass index, and sports injuries in children and adolescents. However, rapid increases in obesity and physical inactivity are likely to be attributable to lifestyle changes, where so-called nurture carries more weight than nature in driving the current pandemic of the aforementioned risk factors [12]. This may imply that children’s lifestyle choices are largely influenced by their parents, especially mothers [13,14]. A recent systematic review identified a 264% increase in the odds of child obesity when mothers are obese during the pre-pregnancy period [13]. Similar findings were observed for physical activity [14], where a higher level of physical activity during the pre-pregnancy period may be related to a lower percentage of body fat in offspring at the age of 5 years [15].

Even though physical activity and body mass index are often interconnected [16], no study has yet examined the associations between maternal physical activity and body mass index with the risk of a childhood sports injury. Although evidence has suggested that the mother–child body mass index and physical activity are associated [14,15], and that childhood obesity and higher rates of sports participation lead to sports injuries [8,9,10,11], it is unknown whether maternal body mass index and physical activity before pregnancy influence the development of childhood sports injuries in their offspring. By examining such associations, public health interventions focused on maternal lifestyle before pregnancy may have protective effects on the reporting of future sports injuries in children.

Therefore, the main purpose of this study was to analyze dose–response associations between maternal pre-pregnancy body mass index and physical activity levels with childhood sports injury rates at the age of 7 years. We hypothesized that body mass index and physical activity in mothers may be longitudinally associated with the incidence of sports injuries reported by their children.

## 2. Materials and Methods

### 2.1. Study Participants

A longitudinal sub-sample was derived from the Czech ELSPAC project, a prospective-birth-cohort study carried out in the Czech Republic. More details about the study were published earlier [17]. In brief, from 1991 to 1992, perinatal information on 7589 children was collected from medical records. From this sample, 5151 mothers from two regions of Brno and Znojmo completed first postnatal questionnaire. A set of questionnaires covering medical and environmental factors was used to collect data. Mothers and their children were followed up for 19 years. Our sub-sample was based on mothers, who completed the questionnaires at baseline, and their children, who reported a sports injury at the age of 7 years. The procedures performed in this study were anonymous and in accordance with the Declaration of Helsinki. Informed consents were obtained from all study participants during each wave of the data collection. An ethics committee approval for all aspects of data collection as well as for the secondary analysis was obtained from the ELSPAC ethics committee. This project has been approved by ELSPAC ethics committee under reference number EL-SPAC/EK/1/2014. Additional details about the study have been published earlier [17].

### 2.2. Sports Injury Assessment in Children at the Age of 7 Years

The primary outcome variable was all sports injuries identified by a “yes” answer to the question: “In the past 12 months have you had at least one sports injury” [4,5,6,8,9]. The reason for including the children at the age of 7 years was because the first sports injuries for both boys and girls are reported in this age group. The injury definition described by van Mechelen et al. [18] was adopted for this study, where an injury is any injury resulting from the participation in physical education classes, sports activities, or leisure-time physical activities with one of the following consequences: the child (i) has to stop the physical activity, (ii) cannot participate in the next planned physical activity, (iii) cannot attend school the next day, or (iv) needs medical attention by physicians or physiotherapists. 

### 2.3. Maternal Physical Activity and Body Mass Index

We used data from questionnaires completed by mothers at the age of 23 years, designed to elicit weekly time spent in physical activity (during leisure time, open-ended answer, and indicated in hours). This measure was used previously to collect data regarding physical activity [19]. Physical activity was used as a continuous variable that enabled the categorization of participants into quartiles. Body height was measured to the nearest 0.1 cm with a portable anthropometer. Body mass was measured to the nearest 0.1 kg using a medical scale. Body mass index was calculated by dividing weight in kilograms with height in square meters (weight (kilograms)/height (square meters)). The international cut points to define underweight (<18.5 kg/m^2^), healthy weight (18.5–24.9 kg/m^2^), and overweight/obesity (≥25 kg/m^2^) were used [20]. The reason for collapsing overweight and obesity into one category was that only 2.3% of mothers were categorized as obese.

### 2.4. Covariates

Independent variables considered as covariates in this analysis were based on potential risk factors including maternal education level being low (primary school), medium (secondary school), or high (higher education); sex of a child (a boy vs. a girl); weekly time spent in physical activity reported by children at the age of 7 years; and child’s weight at birth. Birth weights were categorized as low (<2500 g), medium (2500–3999 g), and high (≥4000 g) [6]. Weekly time spent in physical activity was reported as open-ended answers indicated in hours. For this study, we categorized children into “meeting physical activity guidelines” (participating in ≥420 min of moderate- to vigorous-intensity physical activity per week) or “not meeting physical activity guidelines” (<420 min/week), as prescribed by the World Health Organization [21].

### 2.5. Statistical Analysis

Statistical analyses were performed using Statistical Packages for Social Sciences v.23 (SPSS, Chicago, IL, USA). Basic descriptive statistics are presented as means and standard deviations (SDs) for numerical variables and as frequencies and percentages (%) for categorical variables. The incidence proportion, based on the previous year, was calculated (# injuries/100 participants × 100%) for all sports injuries. To examine the associations between maternal body mass index and physical activity during the pre-pregnancy period and sports injuries reported by children at the age of 7 years, we used generalized estimating equations with binary logistic regression models. To evaluate a reported sports injury, we calculated odds ratios (ORs) and 95% confidence intervals (95% CIs) derived from the analyses of generalized estimating equations. For body mass index and physical activity parameters, “healthy weight” and “the least physically active mothers (<240 min/week)” served as reference categories in all calculations. We ran three logistic regression models. In models 1 and 2, maternal body mass index and weekly time spent in physical activity were used as exposure variables. Each model was adjusted for education level, sex, weekly time spent in physical activity reported by children at the age of 7 years, and childbirth weight. In model 3, maternal body mass index and physical activity were simultaneously input to the model and adjusted for the same covariates. Two-sided *p*-values were used, and significance was set at α < 0.05.

### 2.6. Patient and Public Involvement

Neither patients nor the public were involved in the design or conduct of this study.

## 3. Results

Basic descriptive statistics are presented in Table 1. The majority of mothers included in the analysis had a healthy weight, and they were distributed in similar proportions among each quartile of weekly time spent in physical activity (*p* > 0.05). Approximately one-third of all mothers reported a medium education level. More than half of the children participated in weekly physical activity for ≥420 min, and the majority of them were born with a medium body weight. Of 3273 children aged 7 years, almost 20% of them reported a sports injury in the past 12 months (the incidence proportion for all sports injuries was 63.5%), and the number of sports injuries per 1000 children was 194.

Table 2 shows the associations between maternal body mass index and weekly time spent in physical activity during the pre-pregnancy period and sports injury reports regarding children at the age of 7 years. In model 1, children whose mothers were underweight during the pre-pregnancy period had 74% lower odds of reporting a sports injury at the age of 7 years. Mothers who were overweight/obese during the pre-pregnancy period increased the odds of their children reporting a sports injury at the age of 7 years by 204%. In model 2, a graded, inverse association between maternal pre-pregnancy weekly physical activity and sports injuries was observed: children whose mothers were in higher quartiles had 52%, 59%, and 82% lower odds of reporting a sports injury at follow-up. When body mass index and physical activity were input simultaneously to model 3, mothers being underweight decreased the odds of their children reporting a sports injury at the age of 7 years by 53%, while mothers in the overweight/obesity category increased the odds by 305%. Of note, having a high education level, being a girl, participating in physical activity for ≥420 min/week, and having a low birth weight decreased the odds of children reporting a sports injury at the age of 7 years, while having a high birth weight increased the odds.

## 4. Discussion

The main purpose of this study was to analyze dose–response associations between maternal pre-pregnancy body mass index and physical activity levels and childhood sports injury rates at the age of 7 years. Our main findings are: (i) mothers who were underweight or overweight/obese before pregnancy decreased and increased, respectively, the risk of their children reporting a sports injury at the age of 7 years; (ii) children whose mothers were more physically active in the pre-pregnancy period were less likely to report a sports injury; and (iii) when body mass index and physical activity were simultaneously observed, both underweight status and spending more time in physical activity decreased the risk of children reporting a sports injury at the age of 7 years, while overweight/obesity status increased the risk.

This is the first study examining longitudinal associations between maternal body mass index and physical activity and reported sports injuries; therefore, we compared our results to those in the literature on the same topic in children. Previous cross-sectional studies have shown that obesity may serve as an independent predictor of sports injuries [8,9,10]. Obese youth are more susceptible to sports injuries due to greater forces being absorbed through the joints and soft tissue, especially in a contact sport [22]. Conversely, a longitudinal study by Bloemers et al. [23] showed no significant associations between overweight and obesity status and reported injuries in children aged 9–12 years. However, the relatively small sample lacked the power to specifically identify body mass index as an independent risk factor of sports-related injury due to a small proportion of children participating in sports [23]. From a physiological point of view, obese individuals lack lower-extremity strength and power, which increases the risk of knee- and ankle-related injury [24]. Moreover, impaired neuromuscular and postural control has been confirmed in obese children [25], often accompanied by altered biomechanical loadings of weight-bearing skeletal tissue and an increase in systemic inflammation caused by excess adipose [26]. In general, the most recent systematic review with a meta-analysis showed that of 38 articles studied, 16 identified high adiposity as a significant risk factor for lower-extremity injury [27]. Our results demonstrate that low maternal body mass index has a protective role for sports injuries, which is not in line with the findings in the literature [27,28]. A low body mass index may lead to a higher incidence of bone stress injuries among young female athletes who have a body mass index < 21 kg/m^2^, participate in physical activity ≥ 12 h/week, and have oligomenorrhea/amenorrhea [28]. The discrepancy between studies may be due to different techniques being used to assess adiposity (body mass index in this study vs. densitometry [28]). However, a study by Kemler et al. [29] showed that compared to normal-weight sports participants, the odds of sustaining a sports injury for underweight sports participants are lower by 20% (OR = 0.80, 95% CI 0.56–1.15).

This study provides additional evidence that increased physical activity levels of pre-pregnant mothers lead to a decreased risk of sports injuries being reported by their children at the age of 7 years, which is somewhat contradictory to previous evidence [4,5,6]. A comparison of the results is inherently hampered by differences in study design, population, and injury definition [23]. The small number of longitudinal associations between injury and physical activity found in the literature is often explained by the generic nature of the perceived risk of injury survey items [30]. A prospective 1-year follow-up study showed that the least-active 9–12-year-olds are at an increased risk of sports injuries [23]. Specifically, the highest weekly exposure resulted in a 66% decrease in the odds of reporting a sports injury obtained from a leisure-time physical activity [23]. An Australian 6-year follow-up study found that a higher incidence of sports injuries is associated with lower moderate-to-vigorous and vigorous physical activity in both younger (9 years) and older (11 years) cohorts [30]. The most common leisure activities reported by women include aerobics classes, swimming, and cycling, which is in line with our findings (data not shown). In addition, the guidelines from the 2002 American College of Obstetrics and Gynecology recommend that women participate in recreational activities with a lower risk of injuries and try to avoid the high potential in contact activities, including vigorous team and racket sports [31]. Since parents and their children participate in similar types of sports activities [32], it is not surprising that more time spent in low-risk physical activities reported by mothers in the pre-pregnancy period decreased the odds of their children reporting a sports injury at the age of 7 years.

This study has several strengths. The findings presented in this paper may be used for comparative studies. Central-European populations of children born in the early 1990s grew up under specific socioeconomic and cultural conditions, which were very different from those in the western part of Europe [33]. A population-based, longitudinal design allowed us to establish a certain causality between maternal pre-pregnancy body mass index and physical activity and a child’s sports injury rate at the age of 7 years. Finally, we adjusted for several potential covariates that may have influenced the associations.

Nevertheless, regardless of the significance of the present results, several limitations of the current study should be noted. First, one limitation of the present study is the use of questionnaires to assess physical activity, which typically leads to an overestimation and a larger measurement error [34]. Second, we did not assess whether sports injuries occurred in different modalities, i.e., during physical education classes, leisure-time physical activity, or sports [23]. Another limitation is the inability to examine sport type, mechanism and type of injury, intensity of play, and fitness level as potential effect modifiers between maternal body mass index, physical activity, and the risk of children reporting sports injuries [5,6]. Fourth, the type and intensity of physical activity performed by mothers at baseline were not recorded. Since children are often engaged in the same physical activities as their mothers [32], we speculate that more vigorous-intensity physical activities (including contact sports and sports that include sudden movements) may be prospectively associated with children reporting a sports injury. Fifth, no bone density or blood samples were drawn from the participants, either of which may potentially mediate the associations between body mass index, physical activity, and reports of sports injuries. Finally, more direct measures of body fatness and the collecting of the same data at two or more time points need to be included in future research.

## 5. Conclusions

Pre-pregnancy, underweight mothers decreased the risk of their children reporting a sports injury at the age of 7 years, while overweight/obese mothers increased the risk. There was also a lower risk of a sport-related injury in children whose mothers reported higher levels of physical activity per week. This study adds new information regarding the associations between maternal body mass index and physical activity and sports injuries reported by children. Our data suggest that future sport-related injuries in school-attending children may be prevented by special interventions targeting mothers who are overweight/obese and participate in lower levels of physical activity.

## Figures and Tables

**Table 1:** Descriptive statistics of the study participants.

Study Variables	*n* (%)
Mother at pre-pregnancy	
Age (years), mean (SD)	23.4 (0.5)
Height (cm), mean (SD)	166.3 (6.0)
Weight (kg), mean (SD)	61.2 (12.6)
Body mass index (kg/m^2^), mean (SD)	22.1 (3.3)
Body-mass-index categories	
Underweight	498 (11.1)
Healthy weight	3381 (75.5)
Overweight/obese	597 (13.3)
Weekly time spent in physical activity (min/week)	
<240	475 (24.4)
240–419	430 (22.1)
420–824	500 (25.7)
≥825	541 (27.8)
Education level	
Low	505 (10.6)
Medium	3209 (67.1)
High	1070 (22.4)
Child at the age of 7 years	
Sex	
Boy	846 (49.9)
Girl	849 (50.1)
Sports injury in the past 12 months	
No	2638 (80.6)
Yes	635 (19.4)
Weekly time spent in physical activity (min/week)	
<420	586 (42.1)
≥420	807 (57.9)
Birth weight (g), mean (SD)	3289.6 (594.4)
Birth-weight categories (g)	
<2500	437 (5.9)
2500–3999	6364 (86.6)
≥4000	549 (7.5)

**Table 2:** ORs with 95% CIs for reporting a sports injury at the age of 7 years.

Study Variables	Model 1	Model 2	Model 3
	OR (95% CI)	OR (95% CI)	OR (95% CI)
Mother at pre-pregnancy			
Body mass index			
Underweight	0.26 (0.1–0.68)		0.47 (0.13–0.97)
Healthy weight	Ref.		Ref.
Overweight/obese	2.04 (1.12–3.71)		3.05 (1.36–5.84)
Weekly time spent in physical activity (min/week)			
<240		Ref.	Ref.
240–419		0.48 (0.22–1.00)	0.61 (0.27–1.36)
420–824		0.41 (0.18–0.93)	0.45 (0.19–0.98)
≥825		0.18 (0.07–0.47)	0.22 (0.08–0.59)
Education level			
Low	Ref.	Ref.	Ref.
Medium	0.74 (0.30–1.87)	0.67 (0.27–1.93)	0.62 (0.27–1.91)
High	0.57 (0.37–0.87)	0.44 (0.23–0.82)	0.42 (0.22–0.80)
Child at the age of 7 years			
Sex			
Boy	Ref.	Ref.	Ref.
Girl	0.60 (0.40–0.92)	0.56 (0.30–0.94)	0.57 (0.30–0.99)
Weekly time spent in physical activity (min/week)			
<420	Ref.	Ref.	Ref.
≥420	0.44 (0.29–0.66)	0.58 (0.32–1.10)	0.55 (0.29–1.04)
Birth weight (g)			
<2500	0.59 (0.34–1.00)	0.45 (0.17–1.15)	0.45 (0.17–1.16)
2500–3999	Ref.	Ref.	Ref.
≥4000	1.83 (1.34–2.49)	2.71 (1.72–4.28)	2.53 (1.57–4.09)

Model 1: Examines the associations between maternal pre-pregnancy body mass index and a sports injury reported by their 7-year-old children adjusted for education level, sex, weekly time spent in physical activity, and birth weight. Model 2: Examines the associations between maternal pre-pregnancy weekly time spent in physical activity and a sports injury reported by their 7-year-old children adjusted for education level, sex, weekly time spent in physical activity, and birth weight. Model 3: Examines the associations between maternal pre-pregnancy body mass index and weekly time spent in physical activity simultaneously entered into the model and a sports injury reported by their 7-year-old children adjusted for education level, sex, weekly time spent in physical activity, and birth weight. *p* < 0.05.

## Data Availability

The datasets analyzed during the current study are available upon reasonable request through the website of the Czech ELSPAC project: http://www.elspac.cz/index-en.php (accessed on 13 January 2022).
